# Retrospective analysis of reproductive health indicators in the United Nations High Commissioner for Refugees post-emergency camps 2007–2013

**DOI:** 10.1186/s13031-016-0069-6

**Published:** 2016-03-09

**Authors:** Jennifer Whitmill, Curtis Blanton, Sathyanarayanan Doraiswamy, Nadine Cornier, Marian Schilperood, Paul Spiegel, Barbara Tomczyk

**Affiliations:** Rollins School of Public Health Grace Crum Rollins Building, 1518 Clifton Road, Atlanta, GA 30322 USA; Emergency Response and Recovery Branch, Division of Global Health Protection, Center for Global Health, Centers for Disease Control and Prevention, 1600 Clifton Road, Atlanta, GA 30333 USA; United Nations High Commissioner for Refugees, Case Postale 2500 CH-1211, Genève 2, Dépôt Switzerland

**Keywords:** Reproductive health, Refugees, Health information system, Health indicators

## Abstract

**Background:**

The United Nations Refugee Agency’s Health Information System issues analytical reports on the current camp conditions and trends for priority reproductive health issues. The goal was to assess the status of reproductive health by analyzing seven indicators and comparing them to standards and host country estimates.

**Methods:**

Data on seven indicators were extracted from the database during a seven-year period (2007 through 2013). A standardized country inclusion criterion was created based on the year of country implementation and the percentage of missing reports per camp and year. The unit of analysis was monthly camp reports by year within a country. To account for the lack of independence of monthly camp reports, the variance was computed using Taylor Series Linearization methods in SAS.

**Results:**

Ten of the 23 eligible countries met the inclusion criterion. The mean camp maternal and neonatal mortality rates, except for two country years, were lower than the host country estimates for all countries and years. There was a significant increase in the percent of births attended by a skilled birth attendant (*p* < 0.0001), and 8 of 10 countries did not meet the standard of 100 % for all reporting years. The percent of births performed by Caesarian section (*p* < 0.001), were below the recommended minimum standard for nearly half of the countries every year. There was a significant increase in the percent of women screened for syphilis across years (*p* < 0.0001) and the percent of women who received post HIV exposure prophylaxis (*p* < 0.0001) and 10 % reached the standard for all reporting years, respectively.

**Conclusion:**

Comprehensive, consistent and comparable statistics on reproductive health provides an opportunity to assess progress towards indicator standards. Despite some improvements over time, this analysis confirms that most countries did not meet standards and that there were differences in reproductive health indicators between countries and across years. Consequently, the HIS periodic monitoring of key reproductive health indicators at the camp level should continue. Data should be used to improve intervention strategies.

## Background

Reproductive health (RH) indicators are used by the United Nations High Commissioner for Refugees (UNHCR) and its health partners to ensure resources are correctly targeted to those who need them, respond quickly to public health problems, monitor trends, and evaluate the effectiveness of interventions and service coverage [1]. The RH indicators collected are essential to describe the burden of RH among refugee women of reproductive age. The global distribution of RH indicators can show differences in prevalence between countries or regions that will help inform policy or advocacy but may have very little impact on camp and refugee specific lifesaving interventions. Without timely, and accurate data refugee women could be at increased risk of mortality and morbidity. In general more emphasis needs to be placed on the tools and resources that are needed to collect data to ensure the indicators are of high quality [2].

In January 2013, there were 15.4 million refugees worldwide--approximately 48 % of these refugees were women and girls [3, 4]. As shown in a recent global evaluation of reproductive health (RH) in humanitarian settings from 2012 to 2014, refugee women and children are the most vulnerable to the consequences of displacement [5]. A positive finding in the study showed the number of health proposals with an RH component increased by an average of 10 % per year from 2002 to 2013 [6]. Other positive findings showed significant progress in maternal and newborn health as evidenced by an increase in funding and program evaluations, an increase in funding of gender-based violence (GBV) programs, improvements in mother-to-child-transmission programs and family planning commodities. In contrast, disparities in emergency obstetric care and newborn care services remained unchanged [7] and clinical components of the Minimum Initial Service Package (MISP) were lacking in three settings well beyond the emergency phase [8]. Post-abortion care is still behind compared to other RH interventions due to lack of funding and systematic evaluations. In addition GBV programs showed a lack of prevention efforts against sexual violence and staff trained in the clinical management of rape. Other problem areas found were the inconsistent availability of antiretroviral for HIV and the lack of prevention, testing and treatment of other sexually transmitted infections (STIs). Lastly, an assessment of family planning programs showed a gap in funding and commodities such as intrauterine devices (IUDs), permanent methods and emergency contraception [9]. As humanitarian actors gain experience to solve RH issues, this information will be useful to improve services to populations affected by a crisis.

Since 2006, UNHCR and its health partners have been using a unified health information system (HIS) to monitor refugee public health and HIV programs in camps and urban settings. At the start of 2010, HIS provided services to 1.5 million refugees operating in 18 countries, 85 refugee camps, and through 24 different partners [10]. Past published studies have used the HIS data to evaluate camp nutrition programs, the utilization of outpatient services, the burden of malaria, and under five morbidity and mortality [11-14]. Important contributions of these papers included recommendations to modify nutritional indicators, and the accuracy of population indictor estimates (number of women of reproductive age and children under five). In addition, there were policy and advocacy issues identified such as the need to support equitable and higher quality malaria eradication programs in refugee and host populations. Lastly, through these studies awareness was turned towards the importance of the HIS unified systems utility to analyze and disseminate health information.

To monitor RH services in camps, the HIS uses RH indicators to measure and determine progress in achieving the UNHCR predetermined standards. This study was part of the Interagency Working Group in Reproductive Health Global Evaluation of RH in humanitarian settings. The purpose of this study was to conduct a retrospective review of selected HIS RH indicators of refugees by country to examine trends over time and assess if the indicators meet the UNHCR standards.

## Methods

This analysis used data from RH indicators obtained from UNHCR’s HIS Microsoft Access™ (Redmond, WA) database over a seven-year period (2007 through 2013). Data selected for this analysis were abstracted from the HIS Access database by the HIS supervisor at UNHCR headquarters in Geneva and converted into Excel spreadsheets. The variables included continent, name of country, name of camp, and date of report, and the numerator and denominator values to calculate the indicator. Analysis was conducted from November 2013-July 2014. The US Centers for Disease Control and Prevention (CDC) conducted the data analysis and determined the study was surveillance activity and not human subject research. All analysis was done using SAS software, Version 9.3 of the SAS Institute, Inc.^©^ (Cary, NC) [15].

## Country and camp inclusion criteria

Using HIS data taken from operating refugee camp health facilities from 2006 to 2013, we reviewed HIS data from a total of 23 countries. A country was included in the analysis if it had at least one acceptable camp with monthly reporting data from no later than 2008 and had no more than two unacceptable camps.

A camp was considered acceptable for inclusion into the analysis if it met the following two criteria:

1. Completeness: A camp had completed 90 % of its monthly reports per year (<=10 % missing monthly reports).
2. Total Reporting Months: A camp had at least 6 months of reporting data.

Camps that did not meet the inclusion criteria were not included in the analysis. Data analysis started in 2007 or 2008, depending on the availability of the monthly reports.

## RH indicator inclusion criteria

Fifty-seven RH indicators are included in the HIS system. For this analysis, seven indicators were chosen after screening the RH indicators for usability, plausibility, and relevancy of the reported indicator data. Usability was defined as those indicators identified as high priority by the Inter-agency Working Group on Reproductive Health in Crises [16]. For plausibility, if more than 10 % of the monthly estimates had proportions above 1 prior to cleaning, the data were considered implausible and that indicator was not recommended for analysis. To avoid redundancy, relevancy was determined based on HIS indicators that have been previously analyzed and published. After reviewing the three criteria, the final selection of seven indicators was determined by consensus of RH senior staff at UNHCR and CDC epidemiologists.

The seven indicators chosen for the analysis were the following:

1. Maternal mortality ratio (MMR): Number of pregnancy-related deaths / Total number of live births in a year × 100,000 in a given year.
2. Neonatal mortality rate (NNMR): Number of neonates who died before reaching 28 days of age/Total number of live births × 1,000 in a given year.
3. Proportion of births attended by a skilled birth attendant (SBA) (defined as doctors or midwives who can diagnose and manage obstetrical emergencies and normal deliveries): Number of deliveries attended by skilled birth attendant/Number of deliveries × 100.
4. Proportion of live births performed by a caesarian section: Number of live births performed by Caesarian section/Number of live births × 100.
5. Proportion of antenatal care (ANC) mothers who were screened for syphilis during pregnancy: At the time of delivery, Number of pregnant women who had been screened for syphilis during the antenatal period/Total number of live births × 100.
6. Rate of condom distribution within the entire population: Number of condoms distributed per month/Total population.
7. Proportion of rape survivors who receive HIV post-exposure prophylaxis (PEP) within 72 h of an incident occurring: Number of rape survivors who receive PEP within 72 h of an incident / Total number of rape cases reported.

## Data analysis

### Data cleaning

Within each country, the selected monthly HIS camp indicator variables (numerator and denominator) were assessed for quality and cleaned. Quality was assessed for each of the indicator variables by identifying monthly outliers by camp. For each indicator variable year, outliers were identified for each camp if they varied by more than three standard deviations from the yearly camp mean. If the flagged outlier was considered erroneous after a review, a new value was imputed using average value of the month before and the month after the outlier. Imputing for obvious erroneous values versus setting them to missing allowed us to retain the monthly observations in the data set by giving it a more probable value. Indicators were then created from the cleaned numerators and denominators. The indicators with proportions greater than 1 were recoded to equal 1.

### Statistical analysis

For five of the indicators (syphilis screening, skilled birth attendant, cesarean sections, PEP use and condom distribution), the unit of analysis was the monthly camp reports by year within a country. To take into account the lack of independence of camp monthly reports, the variance was computed using Taylor Series Linearization methods. Specifically, the SAS complex sampling procedures SURVEYMEANS, SURVEYFREQ, SURVEYREG were used for the analysis to account for non-independence. The yearly country indicator point estimates were computed by taking the mean of the monthly camp estimates, and 95 % confidence limits were calculated to estimate precision. To test for linear trends across years within a country, linear regression was used for continuous variables. A *p*-value < 0.05 was considered significant for this analysis.

Due to the small number of the camp deaths per month, the unit of analysis within each country for the two mortality indicators was the aggregated yearly camp reports. To calculate these indicators, the number of maternal or neonatal deaths and the number of live births from each camp was summed on a country level for each year.

Population and live birth data was averaged across years by country. Population data were used to compute the rate of condom distribution within the entire population. We used the number of live births to compute the maternal and neonatal mortality rates, proportion of live births performed by a caesarian section and proportion of ANC pregnant women who were screened for syphilis during pregnancy. For all other indicators, the proportions for each month were created from the camp monthly reported values before the analysis at a year and country level began.

We included maternal and neonatal mortality rates obtained from the World Bank development indicators by host country but not country of origin of the refugee in our analysis. All rates were country aggregate rates. Data for neonatal mortality rates were available for every year of the analysis; however, host country data were only available for maternal morality ratios in 2010 and 2013 [17, 18].

## Results

A total of 23 countries representing 145 camps were evaluated for inclusion in the analysis. Ten countries met the eligibility criteria and were included in the analysis. Nine countries were not included because they did not collect HIS data as early as 2008. Four countries were not included because three or more camps had more than 10 % of their monthly data missing. The ten eligible countries, representing a total population of 268,329, were the following: Bangladesh, Chad, Djibouti, Kenya, Nepal, Tanzania, Thailand, Uganda, Yemen and Zambia. Tanzania and Djibouti were an exception, because they had only one camp reporting during the analysis period but the camp was considered acceptable. Within each country, camps varied in size and number. The number of camps within a country may vary by month because camps may be missing a monthly report or may have opened or closed during the seven year time frame. Three camps were dropped from analysis (2 from Chad, 1 from Kenya) because they had more than 10 % of monthly data missing. The population in each camp could also vary widely during the analysis period (Table 1).

**Table 1:** Summary of population data and camps by country, 2007–2013

Country	No. of camps	No. of months of data	Year HIS began implementation in country	Monthly camp population^a^				Monthly camp live births^b^			
*N* = 10	*N* = 56	*N* = 3566		Mean	Std Dev	Median	IQR^c^	Mean	Std Dev	Median	IQR^c^
Bangladesh	2	154	2007	14,529	3,037	14,510	6,018	43.1	12.8	43	21
Chad	16	994	2007	17,953	7,960	17,362	9,505	57.1	47.6	53.5	49
Djibouti	1	71	2008	13,565	4,031	13,133	8,240	23.1	7.3	22	10
Kenya	5	412	2007	81,723	33,470	48,529	48,529	185.9	83.9	186.5	113
Nepal	2	105	2007	19,291	8,961	18,236	14,854	29.8	17.3	26	19
Tanzania	1	96	2007	57,850	8,262	60,591	12,320	177.8	35.6	174.5	45
Thailand	9	648	2008	15,907	11,474	15,825	10,274	36.4	27.7	33	26
Uganda	13	740	2007	15,333	16,884	8,302	16,380	39.2	51.8	15	51.5
Yemen	3	197	2008	20,314	7,307	21,770	7,965	40.3	17.8	39	17
Zambia	4	149	2008	11,864	4,245	10,687	5,335	49.2	109.2	31	31

^a^Mean of monthly camp data for all years (2007–2013)

^b^Mean of monthly camp live births for all years (2007–2013)

^c^Interquartile range

## Mortality indicators

### Maternal mortality

**Fig. 1:**
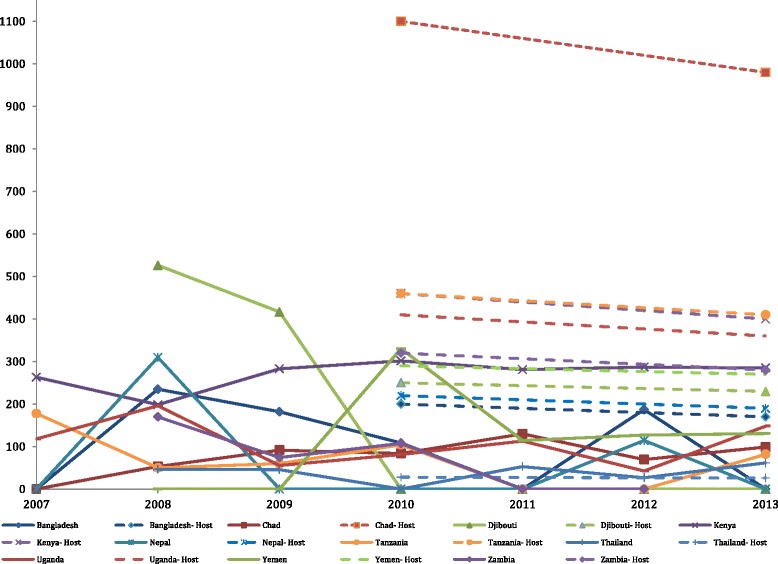
Mean camp maternal mortality ratios (number of maternal deaths per 100,000 live births) by country and year (solid lines), compared with WHO host country maternal mortality ratios (dotted lines)

Figure 1 shows the mean camp MMR for each country and the host country MMR for 2010 and 2013. Three live birth indicator estimates out of 3566 (0.08 %) were imputed for the number of live births and used for calculations in which live births was used; this included the neonatal mortality rate, maternal mortality rate, proportion of births performed by Caesarian section, and proportion of ANC women who were screened for syphilis. There were no imputations for the number of maternal deaths. The mean camp MMR was lower than the host country MMR for all years and all countries, except for Yemen in 2010 and Thailand in 2013. Despite this trend, camp mortality rates varied by country and year. Djibouti reported 526.3 and 416.7 maternal deaths per 100,000 live births in 2008 and 2009, respectively, but did not report a maternal death after 2009. Kenya’s mean MMR was high from 2007 through 2013 with a range across years of 198.5 through 301.5 maternal deaths per 100,000 live births. Chad and Uganda had lower MMRs than many countries in the analysis, ranging between 53.3 and 129.8 and 42.8 and 195.9, respectively. In 2013, the biggest absolute difference between the refugee camp maternal mortality rate and host maternal mortality rate were in Chad (881), Tanzania (−410), and Djibouti (−230). The smallest difference was in Thailand.

### Neonatal mortality

**Fig. 2:**
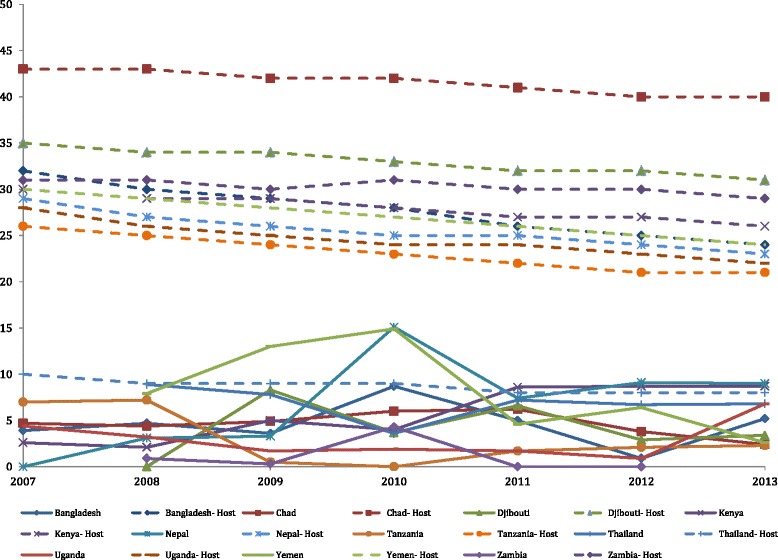
Mean neonatal mortality rates (number of neonatal deaths per 1,000 live births in a year) by country and year (solid lines), compared with WHO host country rates (dotted lines)

The mean yearly camp neonatal mortality rates (NNMR) by country and the WHO NNMR by host country are shown in Fig. 2. No values were imputed for the number neonatal deaths. The mean camp NNMR was lower than the country NNMR in every country for every year. The Nepal camp 2010 NNMR of 15.1 per 1,000 live birth rate was the highest of all country camp data over the course of the study period. Yemen the second highest camp NNMR over the study period, 14.9 in 2010. Zambia’s mean camp NNMR was the lowest of all countries with 0 in 2011 and 2013 and 0.3 and 0.9 in 2008 and 2009 respectively. In 2013, the biggest absolute difference between the refugee camp neonatal mortality rates and host neonatal mortality rates were in Chad (−38), Djibouti (−28), and Yemen (−21). The smallest difference was in Thailand (−1).

## Other reproductive health indicators

The graphs below show the remaining five indicators with the mean and the UNHCR target values for each indicator. The text describes significant trends.

### Proportion of births attended by a skilled birth attendant

**Fig. 3:**
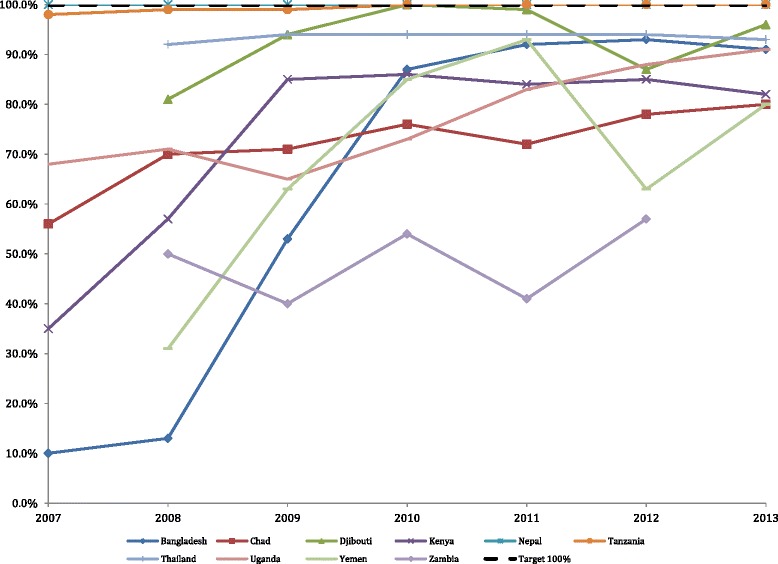
Proportion of births attended by a skilled birth attendant; UNHCR target is 100 % (dotted line)

The plots in Fig. 3 show the yearly average of the monthly camp births attended by a skilled birth attendant for each country along with the UNHCR target number of 100 % coverage. Eight of the 3,566 (0.22 %) skilled attendant birth monthly values were outliers and imputed prior to creating the indicator. The percentage was above 100 % for this indicator in 270 out of 3566 (7.6 %) of the monthly reports. On average most (8 of 10) countries did not meet the target of 100 % for all reporting years. The overall test for a linear trend showed a significant increase over time (*p* < 0.001). The percent of births attended by a skilled birth attendant increased significantly in 6 of 10 countries: Bangladesh (*p* < 0.001), Kenya (*p* = 0.0005), Djibouti (*p* < 0.0001), Tanzania (*p* < 0.0001), Uganda (*p* = 0.0051) and Yemen (*p* = 0.010). Nepal experienced no change, and met the target indicator 100 % of the time over the study period. There was no significant linear trend in 3 of 10 countries: Chad, Thailand, and Zambia.

### Proportion of live births performed by caesarian section

**Fig. 4:**
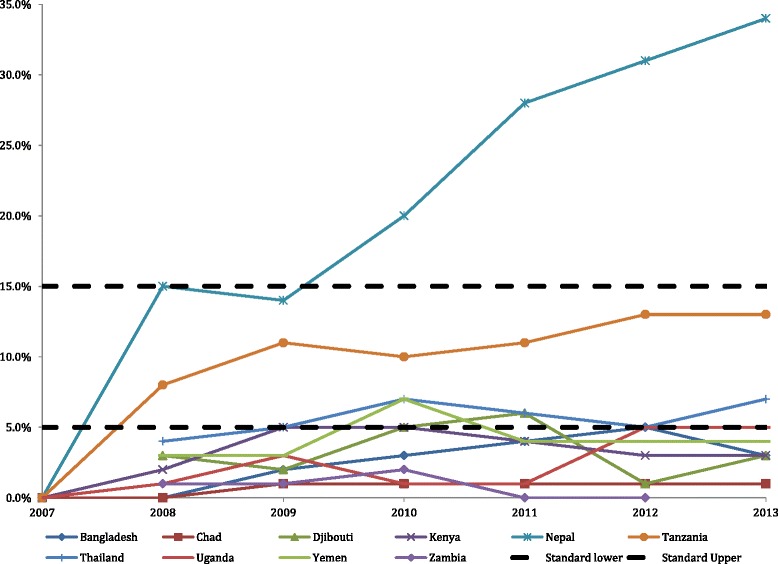
Proportion of live births performed by caesarian section; UNHCR target is between 5 % and 15 %

Figure 4 shows the country-level results for the average percentage of live births performed by caesarian section by year. Nine of the 3566 (0.25 %) monthly values for this variable were imputed before analysis. The WHO recommends to have between 5 % and 15 % of live births delivered through caesarian section. The average monthly caesarian section rates within refugee camps were below the recommended minimum for nearly half of countries every year (4 of 10 countries), and an additional 2 countries only met the minimum once over the study period, Djibouti and Yemen, respectively. However, there was a significant linear trend over time (*p* < 0.001), driven by two countries Nepal and Tanzania, with a larger increase in the percentage of caesarian section deliveries. Seven of 10 countries had a significant increase in proportion of live births performed by caesarian section over the study period: Bangladesh (*p* < 0.0001), Djibouti (*p* < 0.0001), Kenya (*p* = 0.033), Nepal (*p* < 0.0001), Thailand (*p* = 0.029), Tanzania (*p* < 0.0001) and Uganda (*p* = 0.007). Zambia had a significant decrease in percent of caesarian section births over the study period (*p* = 0.038). Chad and Yemen did not have any significant change.

### Percentage of ANC mothers who were screened for syphilis during pregnancy

**Fig. 5:**
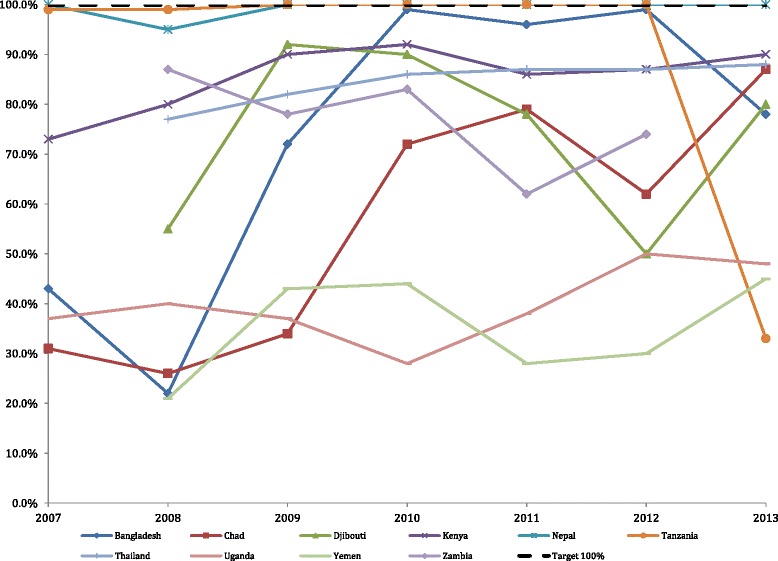
Proportion of women screened for syphilis annually by country. UNCHR target is 100 %

The yearly average of monthly camp syphilis screening by country is presented in Fig. 5. Nine of the 3,566 (0.25 %) monthly camp values for the number of women who received syphilis screening were imputed before the screening indicator was created. There were 731 out of 3,566 (20.5 %) camp monthly reports equal to zero for the antenatal syphilis screening, and 359 out of 3,566 (10.1 %) of the monthly report proportions were over 1. According to the UNHCR target, 100 % of women who come for antenatal care should be screened for syphilis. Only 10 % of all countries for all reporting years reached the standard. There was a significant increase in the yearly percentage of women screened within the refugee camps in this analysis (*p* < 0.001). Five out of 10 countries significantly increased the percentage of ANC mothers screened for syphilis: Bangladesh (p = 0.008), Chad (*p* < 0.0001), Djibouti (*p* < 0.0001), Nepal (*p* < 0.0001), and Thailand (*p* = 0.003). Two countries experienced a significant decrease over the study period, Tanzania (*p* < 0.0001) and Zambia (*p* = 0.007). The remaining 3 did not have a significant change in syphilis screening.

### Rate of condom distribution among the population

**Fig. 6:**
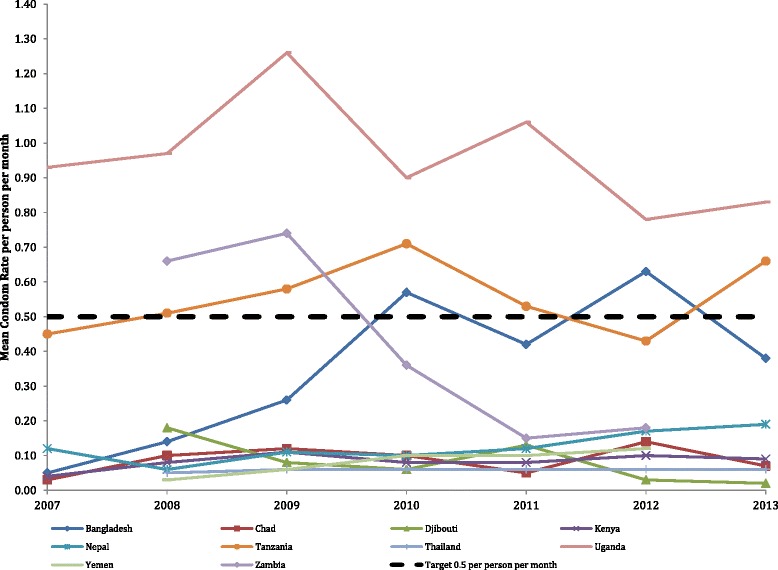
Rate of condom distribution* in the population by year. *Figure depicts percentage (monthly rate*100)

The average yearly rate of camp condom distribution per person per month can be found in Fig. 6. Six variables were used to create the numerator for the condom distribution indicator, and 491 values were imputed of 21,396 (2.3 %). In the denominator, 22 of 3566 (0.62 %) of the total population values were imputed prior to the creation of the condom distribution indicator. There were 658 out of 3,566 (18.5 %) monthly camp values equal to zero for the condom distribution indicator, meaning that during those months there were either no condoms available for distribution and/or no condoms were distributed. Overall, the trend for condom distribution was not significant (*p* = 0.109). There was a significant increase in the rate of condoms distributed in 2 of 10 countries: Bangladesh (*p* < 0.0001) and Nepal (*p* < 0.0001). Three of 10 countries experienced a significant decrease in the rate of condom distribution over time: Djibouti (*p* < 0.0001), Tanzania (*p* < 0.0001) and Zambia (*p* < 0.010), and 5 of 10 had no significant change over time: Chad, Kenya, Thailand, Uganda and Yemen.

### Proportion of reported rape survivors who receive PEP within 72 h of an incident occurring

**Fig. 7:**
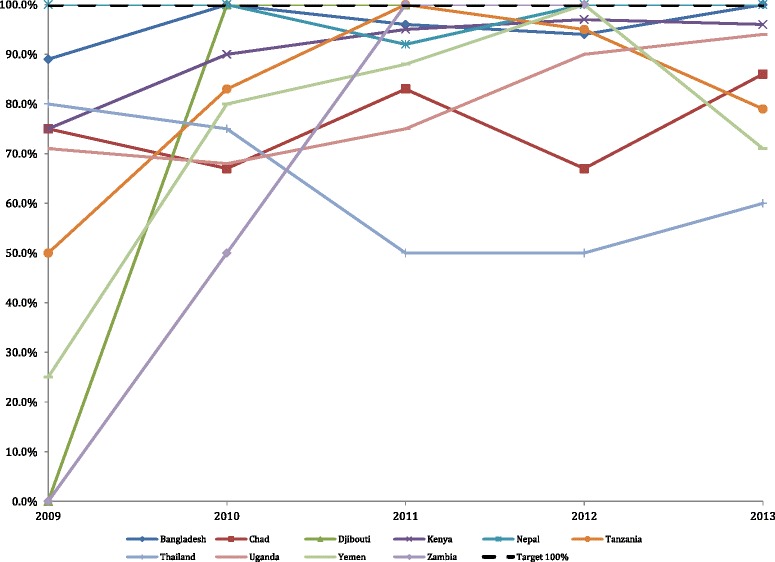
Proportion of rape survivors who received post-exposure prophylaxis (PEP) within 72 h of an incident occurring. UNHCR target is 100 %

Figure 7 displays the proportion of rape survivors who were given PEP within 72 hours of a rape incident. Eight of 3566 (0.22 %) monthly values for women who received PEP and eight of 3566 (0.22 %) of the women who reported rape were imputed before the PEP indicator was created. There were 2971 missing values of 3566 (83.3 %) camp monthly reports for the PEP indicator because there were no reported rapes within camps during those months. Only 10 % of all camps for all reporting years reached the standard, but there was a significant increase in the percentage of women receiving PEP globally (*p* < 0.001). One out of 10 countries had a significant increase over the study period, Uganda (*p* = 0.0001). Six out of 10 countries experienced no significant change over time: Bangladesh, Chad, Kenya, Nepal, Thailand, and Yemen.

## Discussion

This analysis provides an overview of important RH trends over the seven-year study period. For some indicators (births attended by a skilled birth attendant, caesarian section, syphilis screening, and PEP), improvement from 2007 is clear. For other indicators (MMR, NNMR, and condom distribution), more information is needed to explain current trends and why or if improvement is lacking. In several instances standards were not met. Regardless of improvements made in each country for each indicator studied, this analysis demonstrates that more needs to be done to ensure women in refugee camp settings are receiving high quality RH care, as this will decrease morbidity and mortality within the study population. It is often thought that analysis from the HIS is complete, reliable, and of high quality. This analysis demonstrates a need for improved reporting, as many countries were dropped from the study. To close this gap UNHCR and its health partners need to improve strategies and programs to derive maximum benefit from the HIS.

We suspect that HIS is not capturing some of the maternal and neonatal deaths of the camp populations rather than over reporting live births or consistently achieving very low maternal and neonatal mortality in these refugee camp settings. Several previous studies on maternal and infant mortality in refugee camps discussed underreporting of maternal and neonatal mortality [19]; two recent studies that examine the extent of underreporting of neonatal deaths have been completed in camps in Tanzania and Chad [Idowu R, Morof D, Blanton C, Tappis H, Cornier N, Tomczyk B. Using capture-recapture methods and verbal autopsy to understand the incidence of neonatal mortality, stillbirths and live births in UNHCR refugee camps in Chad 2013. Unpublished report.]. Both neonatal and maternal mortality can fluctuate within refugee camps, particularly when influxes of refugees due to an acute emergency occur during a protracted setting. An outbreak such as Hepatitis E that occurred in Dadaab, Kenya disproportionally affected pregnant women [20]. The maternal death audit system is a UNHCR strategy that has been implemented successfully in Dadaab and includes community sensitization to report deaths that occur at home [21]. Other interventions were also included such as improvement of infrastructure, transport, supplies, skilled staffing and mother incentives. This strategy could be replicated in other camps.

Ensuring SBA at delivery is efficacious in contributing to the reduction in maternal and perinatal mortality and helping to reach the post 2015 MDGs 4 and 5 targets [22, 23]. Overall, the significant increase across countries in this study is encouraging. Some refugee camps in this study had a low use of SBAs; this may be due to a lack of SBAs at the facility level. Additionally, the proportions may have exceeded 100 % in some instances because the live births were inaccurately recoded in the monthly reports or because host community women came to deliver and were misclassified as refugees, but more information is needed to determine the root of the inaccuracies. An important consideration for maternity wards at all camps is that they are staffed, 24 h a day, with a professional midwife capable of responding to common obstetric emergencies. It is also important that UNHCR and its partners provide refresher training and supportive supervision, as needed.

Caesarian section was introduced in emergency obstetric care as a lifesaving procedure both for the mother and baby. Overall, there is a positive trend toward meeting the UNHCR standard of caesarean section rates. It is known that the global picture indicates an uneven distribution of caesarian section that shows underuse in low income settings and adequate or even unnecessary use in middle and high income settings [24], and our analysis shows an uneven distribution depending upon the country. The findings from Nepal are counterintuitive since it showed caesarian section rates that would reflect a high income setting. Two studies have shown an inverse association at population level between caesarian section rates and maternal and infant mortality in low income countries where large sectors of the population lack access to basic obstetric care [25, 26], making this indicator an important morbidity and mortality measure. In refugee camps where health care is provided and access to emergency obstetric care may be disproportionately available, this study indicates there are still gains to be made in maternal and infant mortality by increasing access to and use of improved birth technologies, including cesarean delivery. In addition, concerted actions need to be taken to offer timely caesarian section to women who need it and to advocate for a rationale use of caesarian section in camps with a surplus. In Chad and Zambia where caesarian section rates were low more detailed field assessments would help to contextualize the issue and determine the best course of action. Lastly, other important contributing factors that may increase caesarian rates such as previous caesarian sections, and maternal or fetal causes if captured by the HIS could help to interpret this indicator.

Several factors could decrease the syphilis screening rate in refugee camps. For instance, syphilis screening although a routine part of ANC in refugee settings may be missed due to a lack of supplies, equipment and trained staff. Broader ANC may also be lacking, which indirectly leads to women not being screened for syphilis as regularly as they should be. Finally, health care providers may not be prepared in syphilis prevention, and how to prevent re-infection during pregnancy by promoting condom use. Commodities may be in short supply for testing and laboratories require appropriately trained staff for testing [27, 28]. Improvements in UNHCR syphilis screening programs have included implementation of a decentralized program of syphilis screening involving nurses trained in education, counselling and the provision of on-site testing using the Rapid Plasma Reagin test and partner tracing [29].

Condom distribution was inadequate for the majority of camps (6 of 9 camps were less than 50 % in 2013). When looking more directly at the data, condom distribution was sporadic and indicated months with high condom distribution and months with very low to no condom distribution. This indicator may not provide distinct value as a measurement because distribution does not necessarily equate to use, especially where the product is given away free of charge. Refugee populations may also have a varying proportion of children and/or females, making comparisons across refugee countries difficult without adjusting for the number of people who do not need condoms [30]. Logistical difficulties in obtaining and delivering of supplies due to camp location do occur are major obstacles.

Refugee women who have experienced sexual violence should be referred for health services as soon as possible after the incident. The large number of missing data in this analysis points to the fact that either very few women reported a rape in refugee settings or the rapes were reported but not recorded in the HIS, or women are not willing to report a rape as a majority of the monthly reports did not indicate any rapes. The number of rapes reported in each country fall far below global statistics on sexual violence [31]. Legal reforms, protection policy and high quality services available to rape victims have been influential in increasing the likelihood that women will report. Therefore, a multi-sectoral approach is needed in each refugee setting in order to improve services.

There were a number of limitations in this analysis [32]. Underreporting, lack of representativeness, lack of timeliness, and inconsistency of case definitions are four of the most common limitations of many surveillance systems . The HIS has been implemented since 2006 and the quality and completeness of data is known to be somewhat variable during the first months of using the system and may be variable depending on conditions in the individual camps and availability of human resources. A number of countries were excluded from this analysis because the data was too variable. Camps from countries that were not included in the analysis had a higher variance of reporting variability. Some camps rarely reported and other camps within the same country reported fairly regularly. Another limitation is that data quality may be influenced by a number of factors that we did not measure such as newly opened camps versus long term camps, size of camps, availability of RH services and staffing. The inclusion criteria were designed to limit the amount of poor quality data, but they do not ensure that all the monthly reports for 56 camps were of high quality. It also should be noted that we present the average monthly camp estimates by year within a country, but there is variation within a camp and between camps within a country. Sensitive subjects, such as sexual violence may not be reported accurately.

The UNHCR HIS in this study was limited to refugee camps and is facility-based. It may be biased because populations that do not seek care are excluded (survivors of sexual violence, certain RH patients, and deaths occurring outside of health facilities). It is recognized that in refugee camp settings, women may have better access to quality RH care than is available in their country of origin. The Global Evaluation of RH Services for Refugees and Internally Displaced People, conducted in 2004 found that internally displaced persons had worse access to RH services than refugees [33]. Thus we may anticipate that the results may suggest more positive findings than we might find among the surrounding host population or internally displaced persons [34]. Ideally it would have been potentially helpful to have information on how the data were collected in each health facility in each refugee camp in order to improve our understanding of the HIS RH data from this analysis to provide context to the results.

## Conclusion

Many of the refugee RH indicators have been improving over time and most are better than those of host countries, but more can be done to improve the interventions to meet the UNHCR standards. More information is needed on the RH data collection cycle to determine why there was uneven distribution of indicators between countries. In general comprehensive, consistent and comparable statistics on RH provides an opportunity to assess progress towards indicator standards. Despite some improvements over time, this analysis confirms that most countries did not meet standards and that there were differences in RH indicators between countries and across years. Consequently, the HIS periodic monitoring of key reproductive health indicators at the camp level should continue.
